# Risk stratification and predictive value of glucose variability for the development of post-acute pancreatitis diabetes mellitus

**DOI:** 10.3389/fendo.2025.1501530

**Published:** 2025-12-01

**Authors:** Suwan Qian, Yang Liu, Wei Wang, Wenji Ni, Shuna Zhao, Yixin Xu, Ping Gu, Wenkui Yu, Zhihui Tong

**Affiliations:** 1 Department of Critical Care Medicine, Nanjing Jinling Hospital, Affiliated Hospital of Medical School, Nanjing University, Nanjing, China; 2 Department of Critical Care Medicine, Nanjing Drum Tower Hospital, Affiliated Hospital of Medical School, Nanjing University, Nanjing, China; 3 Department of Critical Care Medicine, Affiliated Jinling Hospital, School of Medicine, Southeast University, Nanjing, China; 4 Department of Endocrinology, Nanjing Jinling Hospital, Affiliated Hospital of Medical School, Nanjing University, Nanjing, China; 5 Health Medicine Department, Jinling Hospital, Affiliated Hospital of Medical School, Nanjing University, Nanjing, China; 6 Department of Critical Care Medicine, Jinling Hospital, Nanjing Medical University, Nanjing, Jiangsu, China

**Keywords:** acute pancreatitis, post-acute pancreatitis diabetes mellitus, glucose variability, risk stratification, largest amplitude of glycemic excursions

## Abstract

**Background:**

To assess whether glucose variability (GV) during acute pancreatitis (AP) predicts post-acute pancreatitis diabetes mellitus (PPDM-A), significantly affecting patient life quality.

**Methods:**

This study was performed during 2016–2020 at Jinling Hospital, with a 3-year follow-up for each patient. Cox proportional hazards model was used to evaluate the association of GV with the possibility of developing PPDM-A. Dose-response relationships of GV with the three-year probability of PPDM-A were characterized based on a restricted cubic splines (RCS) model. GV was analyzed to predict the ability for PPDM-A by calculating area under the receiver operating characteristic curves (AUCs).

**Results:**

PPDM-A rates rose from 16% at one year to 27.3% at three years post-AP. Multivariate Cox analysis indicated that the largest amplitude of glycemic excursions (LAGE) exhibited independent association with an increased PPDM-A risk within 3 years (HR = 1.21, 95% CI: 1.05–1.38, P <0.01). RCS results identified optimum LAGE threshold as 5.1, with significantly higher 3-year PPDM-A rates of abnormal LAGE group (LAGE ≥5.1 mmol/L) when compared with normal LAGE group (LAGE <5.1 mmol/L, P <0.001). AUCs for LAGE in predicting PPDM-A incidence in 12, 24, and 36 months were 0.883 (95% CI: 0.862–0.930), 0.916 (95% CI: 0.887-0.945), and 0.926 (95% CI: 0.895-0.948), respectively.

**Conclusions:**

LAGE in hospital stay accurately predicts PPDM-A. Further investigation plays an essential role in determining whether GV-targeting interventions can confer favorable clinical outcomes.

## Introduction

Post-acute pancreatitis diabetes mellitus (PPDM-A) is a specifical diabetes subtype that develops following acute pancreatitis (AP) and is characterized by impaired insulin secretion and glucose metabolism (1–3). It is associated with higher rates of morbidity and mortality, as well as a decreased quality of life (4). The reported incidence of PPDM-A varies widely in different studies, ranging from 5% to 70% (5–8). This variability can be caused by differences in study populations, follow-up durations, and diagnostic criteria. In the Chinese population, recent studies have provided specific epidemiological data: A multicenter retrospective cohort study involving 1,804 patients reported a 6.2% PPDM-A prevalence over a median follow-up of 3.04 years (9); Additionally, Ma et al. constructed a nomogram for predicting PPDM-A in a Chinese cohort of 616 patients, and identified that 20.0% of participants developed diabetes within 3 months of AP onset (10). While traditional risk factors for PPDM-A, such as etiology, obesity, and severity of AP, have been widely investigated, there is still conflicting evidence among a variety of studies (6, 7, 11–16). Therefore, it is essential to identify individuals at high risk for PPDM-A during the recovery phase from an AP episode, which is a vital component of the ‘holistic prevention of pancreatitis’ approach (17).

Several studies showed that in‐hospital hyperglycemia might be a potential indicator for the development of PPDM-A and its associated complications (9, 10, 18). However, these findings have primarily depended on single time point assessments of admission blood glucose or fasting blood glucose levels. This approach, focusing on static blood glucose measurements, may not fully capture the association of dynamic glucose fluctuations with patient prognosis. In recent years, there has been an increasing interest in exploring the impact of glucose variability (GV) on the clinical prognosis of hospitalized individuals. Studies have exhibited significant correlations between increased GV during hospitalization and poorer short- and long-term outcomes. Mendez et al. investigated non-critically ill hospitalized patients (medical/surgical units), finding GV independently predicted prolonged hospitalization (19), while Akirov et al. studied general surgery ward populations (non-cardiac/non-trauma), reporting high GV increased 30-day mortality risk by 78% (20). A study specifically highlighted the predictive value of early-stage GV in plasma glucose levels for the development of diabetes (17). Despite the obtained findings, the specific role of GV in the context of risk stratification and prediction of PPDM-A has not been thoroughly investigated. In addition, the lack of an optimum threshold for GV indices in accurately predicting PPDM-A and effectively stratifying risk has limited the clinical application.

Therefore, a study was performed on Chinese adults following AP by collecting their medical records and conducting telephone follow-ups. This cohort study was used to determine PPDM-A cumulative rates within a 3-year period and to investigate potential risk factors in association with its development. Therefore, this study specifically aimed to (1) propose the novel GV dynamic index and determine optimum threshold of GV indices for enhancing risk stratification, and (2) explore the association of GV indices with the development PPDM-A.

## Methods

### Study design and ethics statement

This cohort study was performed to explore all inpatients who experienced a first attack of AP between January 1, 2016, and December 31, 2020, at the Therapy Center for Severe Acute Pancreatitis (SAP), Jinling Hospital. Data were acquired based on Electronic Medical Record system (AP-Database), with an approval from the Institutional Review Board (2019NZKY-003-03). Analysis was conducted following the specific regulations. Moreover, no informed consents were needed in this retrospective study. To protect privacy, patient information was anonymized and deidentified. Our study was carried out strictly following Declaration of Helsinki and corresponding ethical guidelines.

### Study participants

Subjects enrolled in this study were adults (>18 years) experiencing the initial AP attack. Exclusion criteria were presented as follows: I. Individuals with impaired fasting glucose (IFG, 5.6-6.9 mmol/L) or HbA1c levels ≥5.7% (indicating prediabetes) (21). It should be noted that baseline BMI and HbA1c were not included as risk factors. Most participants had SAP with hypercatabolic states (22), making acute-phase BMI unreliable due to fluid shifts and weight loss (23). HbA1c accuracy may be compromised by anemia, acute kidney injury, or hypoalbuminemia (24). II. Those with other pancreatic injuries, including pancreatectomy, trauma, neoplasms, hemochromatosis, cystic fibrosis, rare genetic diseases, and fibrocalculous pancreatopathy; III. Individuals undergoing severe cardiac/hepatic/renal insufficiency, or those diagnosed with malignancies; IV. Pregnant or postpartum individuals; and V. Individuals with over10% missing data. Defined as missing 3 or more items among 26 core clinical indicators; these indicators include demographic characteristics, AP-related factors, glucose-related metrics, biochemical/inflammatory markers, and complication/prognosis data, detailed in Data Extraction section.

### Data extraction

Demographics (such as sex and age), duration of hospitalization, AP cause and severity, levels of pancreatic enzymes (amylase and lipase), blood glucose levels, serum calcium, interleukin-6 (IL-6), C-reactive protein (CRP), hepatic and renal function markers, lipid profiles, infection status, white blood cell count (WBC), hematocrit (HCT), and platelet count (PLT) were extracted from the AP-Database. Trained professionals were responsible for follow-up visits of subjects after hospital discharge. Follow-up visits were performed through telephone for those who were unable to visit our center. The above indicators constitute 26 core items, with glucose-related metrics (LAGE, SD, CV) merged as one to avoid overcounting. An individual is deemed to have ‘over 10% data missingness’ if missing 3+ of these 26 items and thus excluded.

### Definitions and classifications

Based on Atlanta classification after revision, AP was diagnosed according to two among these three criteria (25): (1) sudden severe and persistent upper abdominal pain usually radiating to the back. (2) serum lipase and/or amylase levels ≥ 3-fold upper limit of normal, and (3) representative imaging results consistent with AP on abdominal ultrasound, contrast-enhanced CT, and MRI. AP severity was classified into mild, moderate and severe following revised Atlanta classification (25). Subjects of study were categorized into mild AP when they did not have systemic or local complications or organ failure. Moderate AP was defined in patients developing systemic or local complications without persistent organ failure (< 48 h). Severe AP was defined in patients with persistent single/multiple organ failure (> 48h). Consistent with 2021 American Diabetes Association (ADA) standards, PPDM-A was defined as new-onset diabetes following AP (>3 months) without underlying diabetes prior to AP attack (26).

### Definition of GV indices and abnormal GV

The GV indices, including Standard Deviation of Blood Glucose (SDBG), Largest Amplitude of Glycemic Excursions (LAGE), and Coefficient of Variation (CV) are employed to assess blood glucose level variability among people with diabetes. Firstly, serum glucose levels measured in the course of disease were detected in all patients. Subsequently, SDBG was defined as arithmetic SDBG values. CV was defined as the SDBG to average blood glucose level ratio. LAGE was calculated as the difference between the highest and lowest blood glucose values (mmol/L) recorded during a 24-hour observation period, serving as a key metric of glycemic variability independent of average glucose concentrations (27). In healthy adults with normal glucose tolerance, a LAGE value of under 5.7 mmol/L is considered within the normal physiological range (28).In our study, frequent capillary blood glucose (CBG) measurements (finger-prick) offer a clinically feasible method for LAGE calculation. CBG was measured every 2 hours and LAGE was calculated as the difference between the maximum and minimum glucose value across a 24-hour period (requiring 12 measurements). LAGE values were averaged across three monitoring days to enhance reliability. To identify the GV index most strongly associated with the occurrence of PPDM-A within three years, least absolute shrinkage and selection operator (LASSO) regression were used. This model allowed us to screen the most relevant GV index from the above-mentioned indices. To further analyze the optimum thresholds of these GV indices, RCS regression was used for evaluating dose-response associations of indices of GV with the 3-year probability of PPDM-A. Regression involved possible confounding factor adjustment at 5, 25, 75, and 95th percentiles of GV indices. The optimum threshold was GVs value that corresponded to lower limit of HR 95% CI > 1. Therefore, GV value ≥ threshold was deemed as having aberrant GV; otherwise, they were classified as having normal GV.

### Statistical analyses

A Shapiro–Wilk test was used to evaluate continuous variables were normally distributed. Based on the findings, continuous variables were indicated by mean and standard deviation or median and interquartile range. Normally-distributed data were compared by t-test, whereas non-normally-distributed counterparts were compared by adopting Mann-Whitney test.

Hazard ratio (HR) having 95% confidence intervals (CI) was calculated by using univariate and multivariate Cox proportional hazards regression models. Variables satisfying P < 0.05 through univariate regression and clinically relevant characteristics were selected as candidate risk factors. LASSO regression satisfying P < 0.05 was used to screen best-fit covariates in multivariate regression (**Supplementary Figure 1**). Correlation heatmaps were plotted to test multi-collinearity among variables in the multivariate COX regression models (**Supplementary Figure 2**).

In addition, 3-year PPDM-A incidence was evaluated by drawing Kaplan–Meier survival curves, which was compared with Logrank test in abnormal and normal LAGE groups. Area under receiver operating characteristic (ROC) curves (AUC) was calculated to assess the PPDM prediction ability. Delong test was utilized to compare ROC curves.

Subgroup analyses stratified by different factors, including age (≤50, >50 years), gender, individuals with or without walled-off necrosis(WON), and those with recurring AP, were performed. Moreover, the association of abnormal GV with the above subgroup variables was analyzed, and the correlation of abnormal GV with 3-year PPDM-A incidence was analyzed in every subgroup.

P < 0.05 (two-tailed) represented statistical significance. R Programming Language (version 4.0.3) and STATA (version.15.0) were used for statistical analyses. R packages “ggrcs” [0.2.4 version] and “rms” [6.2–0 version] were utilized to visualize and analyze the RCS model; “survival” [3.2–11 version] was used in survival analysis; “timeROC” [0.4 version] was applied to estimate time-dependent ROC curve and AUC); whereas “forestplot” [2.0.1 version] was used to visualize forest plots during sensitivity analysis.

## Results

**Figure 1:**
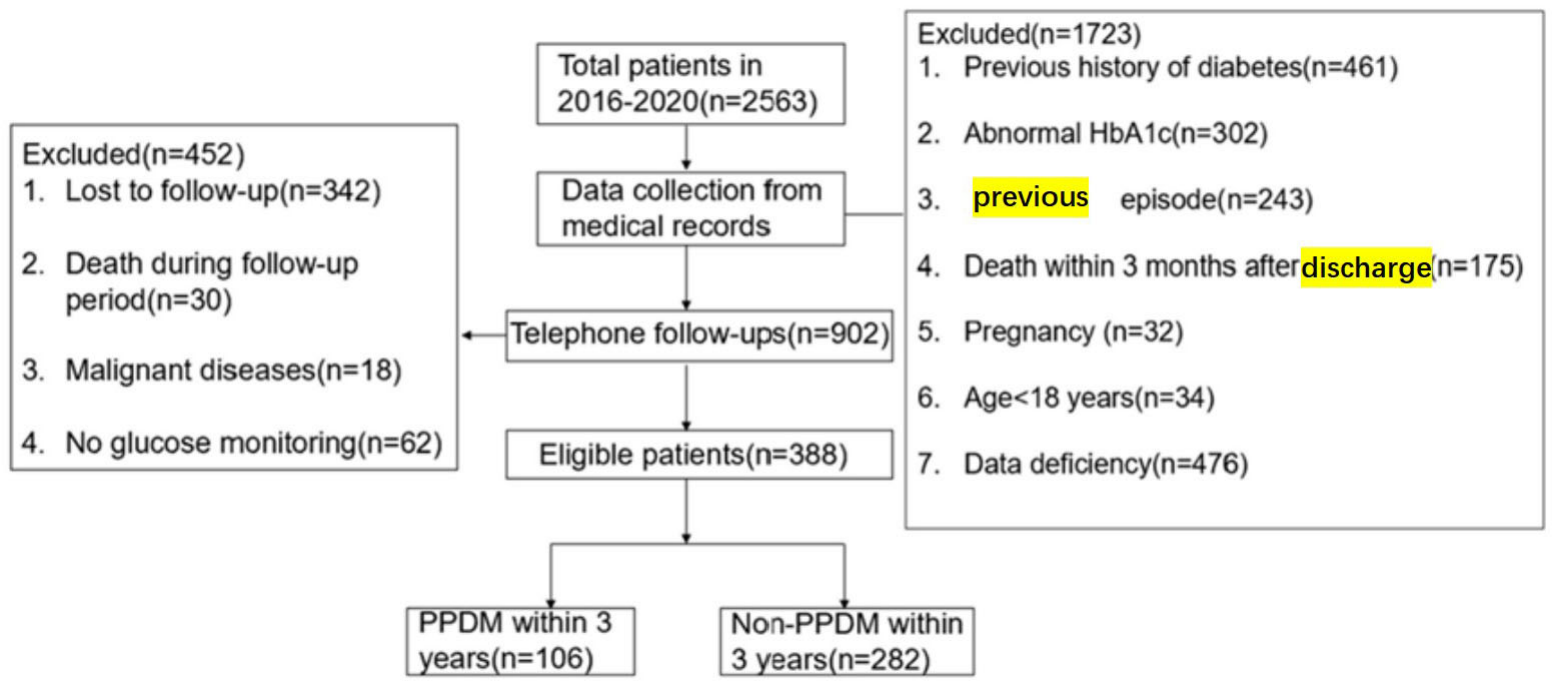
Flow-chart of the study group selection process.

Finally, totally 388 subjects satisfying the eligibility criteria were enrolled for analysis. DM was observed in 27.3% (106/388) of patients after the onset of AP(**Figure 1**). PPDM-A developed in 62, 91, and 106 patients at 1, 2, and 3 years after the onset of AP, with cumulative rates of 16%, 23.5%, and 27.3%, respectively.

### Patient features

**Table 1** displays patient demographic and clinical data, with 388 cases being included in this study. Based on diabetes, patients were classified into two groups. There were no significant differences in terms of gender and age between the two groups. However, individuals with PPDM-A showed longer hospital stays (12 (6–39) *vs* 7 (4–18), p<0.001), elevated levels of uric acid (UA), triglycerides (TG), procalcitonin (PCT), amylase, IL-6, CRP, admission blood glucose (ABG), LAGE, coefficient of variation (CV), and HCT (all P < 0.05), as well as a higher severity of AP (P = 0.006) in relative to non-diabetic patients. Individuals with PPDM-A had a higher incidence of hyperlipidemic AP (p<0.001) than those who had normal glucose levels, and they also exhibited a higher AP recurrence rate, as well as a higher occurrence of Wall-off necrosis (WON), acute kidney injury (AKI), acute respiratory distress syndrome (ARDS), and infected pancreatic necrosis (IPN) when compared with individuals without diabetes (all P < 0.05).

**Table 1:** Patient demographics and clinical characteristics.

Characteristic	AP without DM (n=282)	PPDM-A (n=106)	P value
Age [median (IQR)], years	45 (36, 54)	44(35, 52)	0.62
Male, n(%)	178 (63)	72 (68)	0.378
Hospital stay[median (IQR)], days	7(4, 18)	12(6, 39)	<0.001
Severity, n(%)			0.006
Mild	52 (18)	6 (6)	
Moderately severe	71 (25)	27 (25)	
Severe	159 (56)	73 (69)	
Etiology, n(%)			<0.001
Biliary	161 (57)	36 (34)	
Hyperlipidemic	94 (33)	62 (58)	
Alcoholic	6 (2)	4 (4)	
Idiopathic	21 (7)	4 (4)	
ABG [median (IQR)], mmol/L	6.9 (5.7, 8.5)	10.5 (7.9, 13.9)	<0.001
LAGE [median (IQR)], mmol/L	3.9 (2.3, 5.8)	10.4 (8.2, 13.9)	<0.001
SD_BG_	1.94 (1.13, 2.74)	5.18 (4.11, 6.25)	<0.001
CV%	28 (17, 36)	47 (38, 58)	<0.001
IL-6 [median (IQR)], ng/L	55 (25, 151)	136 (83, 270)	<0.001
PCT [median (IQR)], ug/L	0.4 (0.2, 1.3)	0.5 (0.2, 1.9)	0.013
Amylase [median (IQR)], U/L	87 (41, 228)	57 (35, 140)	0.046
Lipase [median (IQR)], U/L	302(64, 674)	243(72,631)	0.351
LDH [median (IQR)], U/L	671 (460, 1,162)	712 (426, 1,297)	0.336
TG [median (IQR)], mmol/L	1.72 (1.14, 2.92)	2.58 (1.56, 5.41)	<0.001
TC [median (IQR)], mmol/L	3.26 (2.44, 4.16)	3.38 (2.55, 4.89)	0.145
Albumin [median (IQR)], g/L	30.2 (26.5, 34.6)	31.2 (28.0, 34.8)	0.175
Cr [median (IQR)], umol/l	53 (41, 71)	55 (42, 77)	0.179
BUN [median (IQR)], mmol/l	4.3 (3.0, 6.5)	4.9 (3.2, 7.7)	0.189
UA [median (IQR)], umol/L	204 (136, 288)	241 (162, 327)	0.010
WBC, ×109/L	10.5 (7.6, 14.1)	11.4 (8.1, 15.0)	0.171
PLT, ×106/L	193 (143, 255)	214 (151, 299)	0.032
HCT			
CRP [median (IQR)], mg/L	114 (52, 185)	210 (135, 263)	<0.001
WON, n(%)	154 (55)	78 (74)	<0.001
IPN, n(%)	85 (30)	46 (43)	0.014
ARDS, n(%)	65 (23)	45 (42)	<0.001
AKI, n(%)	54 (19)	32 (30)	0.02
Recurrence, n(%)	30 (11)	22 (21)	0.009

Values are presented as means ± standard deviation (SD), median (IQR) or frequencies (percentages). *Statistically significant (P < 0.05) for patients with PPDM-A vs. normoglycemia.

CRP, C-reactive protein; LDH, lactate dehydrogenase; Cr, creatinine; BUN, blood urea nitrogen; UA, uric acid; TG, triglyceride; TC, total cholesterol; ABG, admission blood glucose; LAGE, Largest amplitude of glycemic excursions; CV, coefficient of variation; PCT, procalcitonin; WON, Wall-off necrosis; IPN, infected pancreatic necrosis; ARDS, acute respiratory distress syndrome; AKI, acute kidney injury.

The meaning of the bold values is statistical significance (P < 0.05) for patients with PPDM-A vs AP without DM.

### Relationship of GV indices and clinical features to PPDM-A incidence

Univariate Cox regression analysis indicated that elevated levels of ABG, LAGE, SDBG, and CV, as well as a higher recurrence rate of AP, occurrence of WON, IPN, ARDS, and AKI, longer hospital stays, elevated IL-6, TG, Cr, BUA, HCT, PLT, and CRP, as well as the severity and etiology of AP, were all associated with an increased risk of 3-year probability of PPDM-A (**Table 2**).

**Table 2:** Univariate and multivariate association of LAGE and 3-year incidence of PPDM-A in COX regression model.

Variables	HR	[95% CI]	P1	aHR	[95% CI]	P2
ABG	1.16	1.13-1.19	p<0.001	-	-	
LAGE	1.25	1.21-1.29	p<0.001	1.27	1.22-1.32	p<0.001
SD	1.47	1.40-1.55	p<0.001	-	-	
CV%	1.06	1.05-1.07	p<0.001	-	-	
Severity	reference	-	-	-	-	
	3.05	1.26-7.38	0.014	-	-	
	3.45	1.50-7.92	0.004	-	-	
Etiology	reference	-	-	-	-	
	2.54	1.68-3.83	p<0.001	-	-	
	2.72	0.97-7.65	p=0.058	-	-	
	0.84	0.30-2.37	p=0.748	-	-	
WON	2.08	1.35-3.21	p=0.001	-	-	
ARDS	2.09	1.42-3.07	p<0.001	-	-	
AKI	1.68	1.11-2.54	p=0.014	-	-	
Age	1.00	0.98-1.01	p=0.516	-	-	
LOS, days	1.01	1.01-1.02	<0.001	-	-	
recurrence	1.90	1.19-3.04	0.007	-	-	
Gender, male	1.19	0.79-1.78	0.411	-	-	
IL-6, per 10 ng/L	1.01	1.01-1.02	p<0.001	1.01	1.00-1.01	0.004
PCT, ug/L	1.00	0.99-1.01	0.553	-	-	
Amylase, per 10 ng/L	0.99	0.99-1.00	0.253	-	-	
LDH, per 10 U/L	1.00	1.00-1.01	0.053	1.01	1.00-1.01	<0.001
TG, mmo/L	1.08	1.05-1.11	<0.001	-	-	
TC, mmo/L	1.00	0.98-1.03	0.926	-	-	
Albumin, g/L	1.02	0.99-1.05	0.242	-	-	
Cr, per 10	1.02	1.01-1.04	0.002	-	-	
BUN, mmol/L	1.04	1.02-1.06	<0.001	-	-	
UA, per 10 ng/L	1.02	1.01-1.04	<0.001	-	-	
WBC, ×109/L	1.02	0.99-1.06	0.129	-	-	
HCT	0.06	0.00-0.92	0.044	-	-	
PLT, per 10*109/L	1.02	1.01-1.04	0.003	1.02	1.01-1.04	0.003
CRP, per 10 mg/L	1.08	1.05-1.10	<0.001	1.04	1.02-1.07	<0.001

HR Hazard ratio, aHR adjustive Hazard ratio, P1 p-value for univariate analysis, P2 p-value for multivariate analysis, CRP, C-reactive protein; LDH, lactate dehydrogenase; Cr, creatinine; BUN, blood urea nitrogen; UA, uric acid; TG, triglyceride; TC, total cholesterol; ABG, admission blood glucose; LAGE, Largest amplitude of glycemic excursions; CV, coefficient of variation; WON, Wall-off necrosis; IPN, infected pancreatic necrosis; ARDS, acute respiratory distress syndrome; AKI,acute kidney injury. - denoted variables that were screened out by the least absolute shrinkage and selection operator (LASSO) regression.

Consistent with the -2log-likelihood and binomial family type measure, LASSO regression was conducted out with R software through 10-fold K cross-validation to centralize and normalize those involved variables and to select the optimum lambda value. “Lambda.lse” provides the model with least independent variables yet high performance. Finally, those 5 potential risk factors from the candidate variables were selected by LASSO regression, including LAGE, IL-6, PLT, CRP, and LDH. In addition, the most significant features selected by LASSO were adopted for multivariate Cox proportional hazards analyses.

The multivariate Cox regression analysis identified the higher levels LAGE (aHR = 1.21, 95% CI: 1.05–1.38, P <0.001), CRP (aHR = 1.04, 95% CI: 1.02–1.07, P = 0.001), IL-6 (aHR = 1.01, 95% CI: 1.00–1.01, P = 0.004), LDH (aHR=1.01, 95% CI: 1.00–1.01, P <0.001) and PLT (aHR = 1.02, 95% CI: 1.01–1.04, P = 0.003) as independent prognostic factors for PPDM-A.

**Figure 2:**
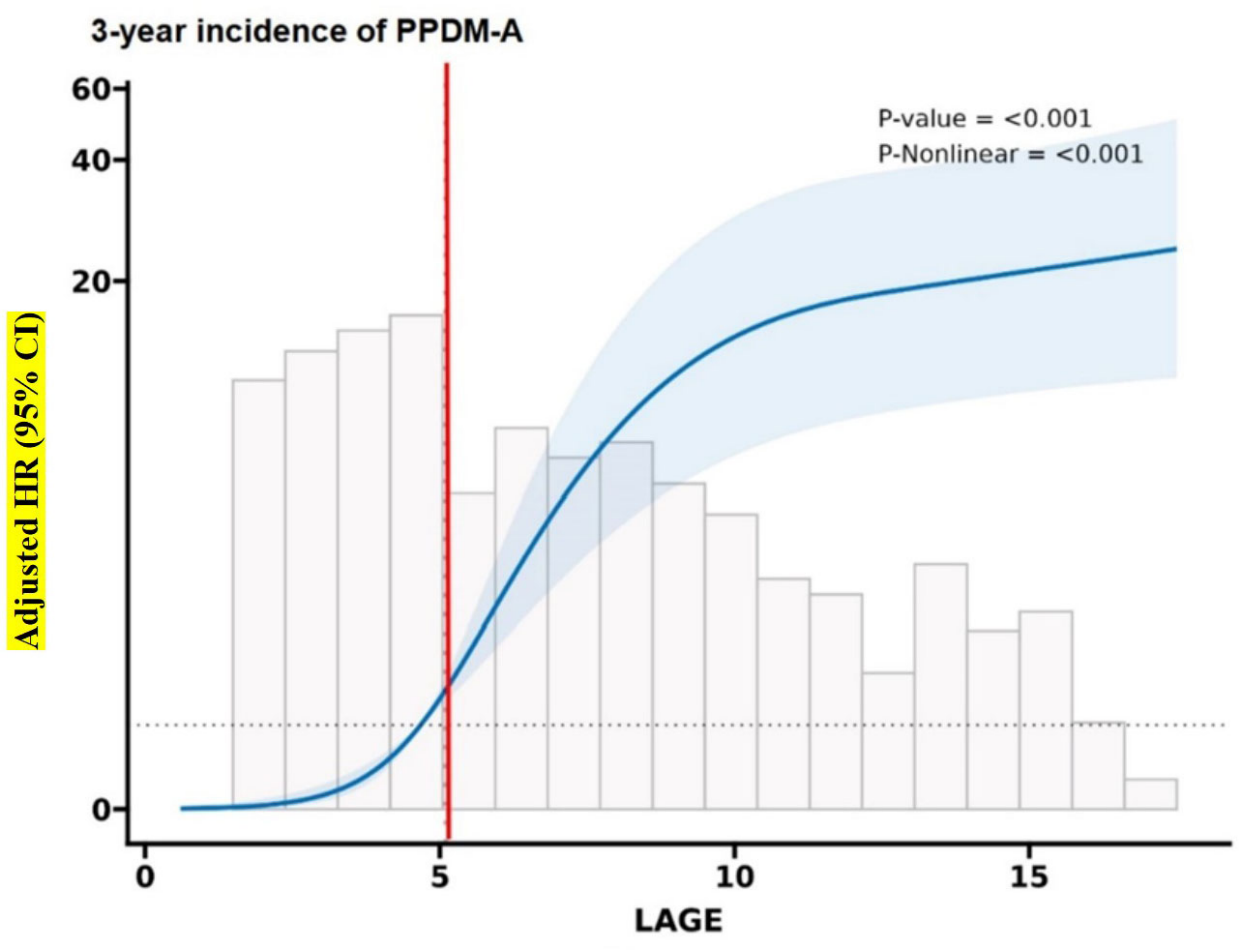
Association between LAGE and the incidence of PPDM-A within 3 years in AP patients. Models are adjusted for etiology, CRP, Plt, and LOS. The blue line and light blue area represent the estimated hazard ratio and its 95% confidence interval. The red vertical line represents LAGE = 5.1 mmol/L. CI confidence intervals, HR hazard ratio.

Multivariate RCS analyses adjusted for IL-6, CRP, LDH, and platelet count suggested that LAGE was correlated with 3-year probability of PPDM-A in AP cases (**Figure 2**). The dose–response curve morphology exhibited monotonic increase. The lower limits of 95% CI associated with HR for 3-year probability of PPDM-A were > 1 at LAGE ≥ 5.1 mmol/L. Therefore, the optimum threshold was LAGE = 5.1 mmol/L. LAGE < 5.1 mmol/L and LAGE ≥ 5.1mmol/L were deemed as normal and abnormal LAGE, respectively.

In the normal LAGE group, increasing LAGE levels were not significantly correlated with the 3-year likelihood of developing PPDM-A. However, for abnormal LAGE group, the increasing LAGE levels were related to a higher PPDM-A risk over 3 years.

### Clinical outcomes for both LAGE groups

Participants were categorized into normal (205, 52.8%) or abnormal LAGE group (183, 47.2%). The baseline clinical characteristics showed significant variances between the two groups. Compared to the normal LAGE group, patients with abnormal LAGE (≥5.1 mmol/L) had significantly longer hospital stays, higher rates of severe acute pancreatitis, more frequent hyperlipidemic etiology, and increased complications such as ARDS, AKI, and walled-off necrosis (**Table 3**). Moreover, patients with abnormal LAGE exhibited a higher incidence of PPDM-A within 3 years compared with those with normal LAGE. (**Supplementary Figure 3**, Log-rank P < 0.001).

**Table 3:** Baseline clinical characteristics of patients stratified by the optimal cutoff point of LAGE.

Characteristic	Normal LAGE (205)	Abnormal LAGE (183)	P-value
Age, (mean ± SD), years	46 ± 14	45 ± 12	0.535
LOS, (mean ± SD), years	12 ± 16	27 ± 33	<0.001
IL-6, (mean ± SD), ng/L	118 ± 171	200 ± 326	0.002
PCT, (mean ± SD), ug/L	3.2 ± 18.6	3.7 ± 11.2	0.757
Amylase, (mean ± SD), U/L	193 ± 260	177 ± 263	0.567
LDH, (mean ± SD), U/L	787 ± 580	1,259 ± 1,284	<0.001
TG, (mean ± SD), mmo/l	2.41 ± 2.58	4.09 ± 5.59	<0.001
TC, (mean ± SD), mmo/l	3.54 ± 1.49	4.48 ± 9.73	0.200
Albumin, (mean ± SD), g/l	31.3 ± 6.2	30.9 ± 6.2	0.518
Cr, (mean ± SD), umol/l	66 ± 69	91 ± 101	0.006
BUN, (mean ± SD), mmol/l	5.2 ± 3.8	6.8 ± 7.5	0.010
UA, (mean ± SD), umol/l	225 ± 108	249 ± 147	0.077
WBC (×109), mean ± SD	11.5 ± 5.4	11.9 ± 5.8	0.524
HCT, mean ± SD	0.34 ± 0.07	0.33 ± 0.07	0.153
Plt(×109), mean ± SD	218 ± 111	226 ± 117	0.523
CRP, (mean ± SD), mg/L	121 ± 83	166 ± 92	<0.001
Severity			<0.001
Mild	49 (24)	9 (5)	
Moderately severe	52 (25)	46 (25)	
Severe	104 (51)	128 (70)	
Etiology			<0.001
Biliary	125 (61)	72 (39)	
Hyperlipidemic	62 (30)	94 (51)	
Alcoholic	6 (3)	4 (2)	
Idiopathic	12 (6)	13 (7)	
WON, n(%)	106 (52)	126 (69)	<0.001
IPN, n(%)	59 (29)	72 (39)	0.028
ARDS, n(%)	34 (17)	76 (42)	<0.001
AKI, n(%)	29 (14)	57 (31)	<0.001
Recurrence, n(%)	20 (10)	32 (17)	0.026
Male, n(%)	129 (63)	121 (66)	0.512
PPDM-A, n(%)	4 (2)	102 (56)	<0.001

Data are presented as n (%) or mean ± SD. CRP, C-reactive protein; LDH, lactate dehydrogenase; Cr,creatinine; BUN, blood urea nitrogen; UA, uric acid; TG, triglyceride; TC, total cholesterol; ABG, admission blood glucose; LAGE, Largest amplitude of glycemic excursions; CV, coefficient of variation; WON, Wall-off necrosis; IPN, infected pancreatic necrosis; ARDS, acute respiratory distress syndrome; AKI,acute kidney injury; LOS length of hospital stay.

### Predicting ability of LAGE for PPDM-A incidence

**Figure 3:**
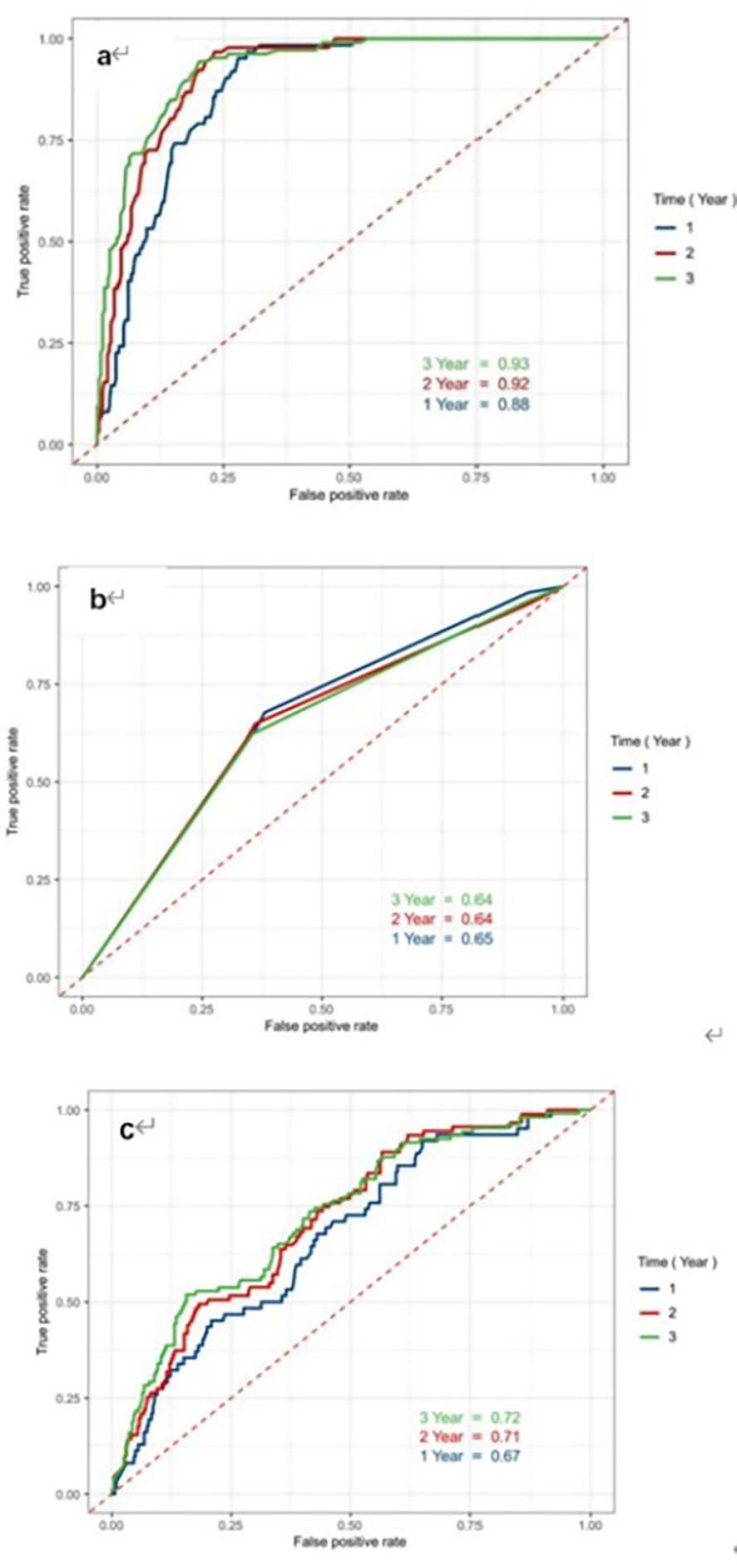
Receiver Operating Characteristic curves of indicators LAGE, CRP and etiology to predict the risk of developing PPDM-A within 1 year, 2 years and 3 years. **(a)** LAGE; **(b)** CRP; **(c)** etiology of AP; ROC receiver operating characteristic, AUC the area under the receiver operating characteristic curves.

Differences in predicting ability of LAGE, and the etiology of AP and IL-6 for PPDM-A at different time points were compared by using time-dependent ROC curve analysis. The AUCs of the LAGE in determining 1-, 2-, 3-year PPDM-A risk were 0.883 (95% CI: 0.862–0.930), 0.916 (95% CI: 0.887-0.945), 0.926 (95% CI: 0.895-0.948), separately (**Figure 3a**). The CRP discrimination was 0.66 (95% CI: 0.575–0.735), 0.70 (95% CI: 0.633–0.765), and 0.69 (95% CI: 0.636–0.752) in determining 1-year, 2-year, 3-year probability of PPDM-A respectively. (**Figure 3b**). In previous studies, the etiology of AP has been regarded as the risk factor related to DM occurrence after AP, and AUC of the etiology of AP for 3-year incidence of PPDM-A was 0.60 (95% CI: 0.544-0.657) (**Figure 3c**).

### Sensitivity analysis on the correlation of abnormal LAGE with 3-year PPDM-A incidence

**Figure 4:**
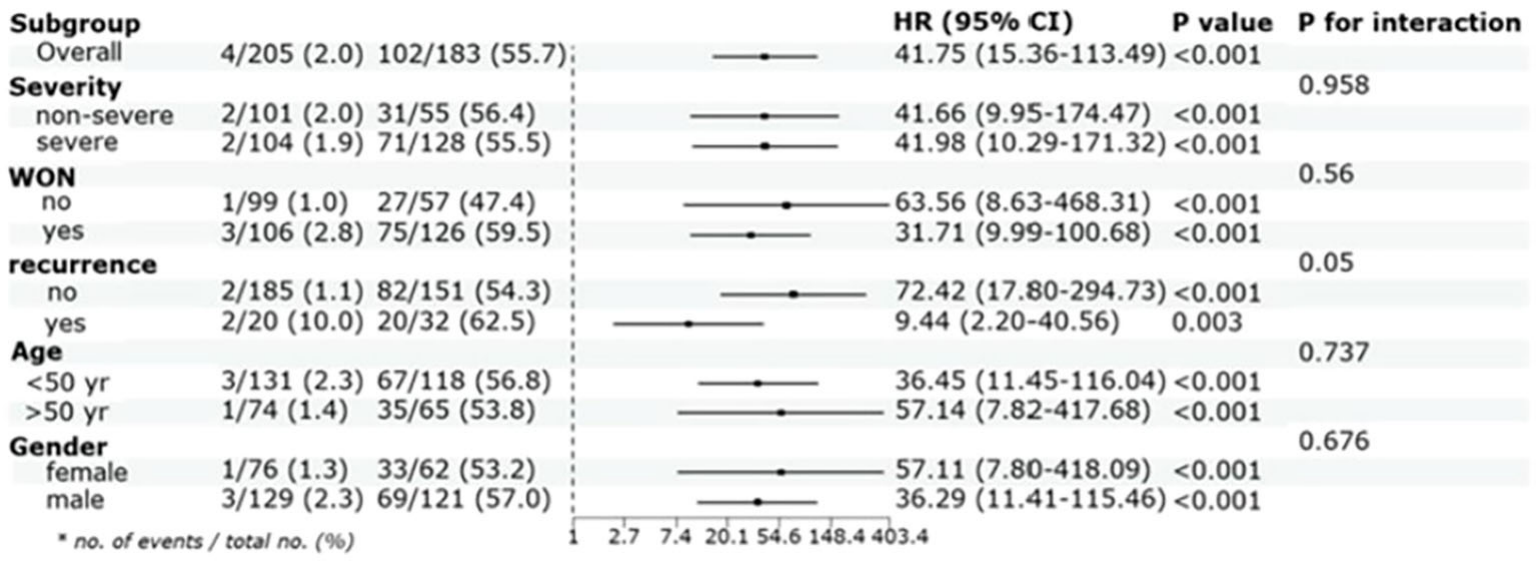
Cox proportional hazards analysis evaluating LAGE in various stratifications. HR was evaluated by abnormal LAGE and normal LAGE and were adjusted for CRP, IL-6, PLT and LDH, HR:hazard ratio, CI:confidence interval, yr: year.

Participants of abnormal LAGE group exhibited the significantly increased PPDM-A risk over 3 years compared with those in normal LAGE group, even when some confounders were adjusted (aHR =41.75, 95% CI:15.36–113.49, P < 0.001). Moreover, abnormal LAGE was consistently associated with an elevated possibility of developing PPDM-A within 3 years across various subgroups, including patients aged >50 and ≤50 years, men and women, participants with/without WON, and those with recurring AP. It is vital to emphasize that even in patients with non-severe AP, the abnormal LAGE group still exhibited a relatively higher incidence of PPDM-A over 3 years (HR = 41.66, 95% CI:9.95–174.47) (**Figure 4**).

## Discussion

Individuals with PPDM-A confront a significant risk to their health and lives due to frequent hypoglycemia episodes and complications (1, 29–31). Our study was aimed at providing a more comprehensive understanding of PPDM-A, building on our 2016 research that had limitations in scope and definition (32, 33). It was found that the prevalence of PPDM-A increased from 16% at one year to 27.3% at three years after AP onset, which is higher than the 23% cumulative incidence reported in meta-analyses (6, 34). This discrepancy may be caused by methodological differences and regional variations (12, 16).

In this study, we innovatively demonstrated the predictive value of GV for PPDM-A, identifying LAGE = 5.1 mmol/L as optimum threshold in risk stratification. Our findings were strong in different subgroups, even for non-severe AP patients. Elevated glucagon and pancreatic polypeptide levels, alongside decreased ghrelin and peptide YY responses, are characteristics of PPDM-A and could serve as potential biomarkers (35). Our research builds on the known association of GV with mortality and diabetes, demonstrating it as the significant risk factor related to PPDM-A. Different from previous studies concentrating on early glucose fluctuations, our analysis considered the entire disease course, from admission to discharge, providing a more comprehensive assessment of GV’s predictive value for PPDM-A development (19, 20, 36, 37).

Abnormal blood glucose levels in hospitalization are related to the higher PPDM-A risk (16–18). Univariate analysis suggested that the glucose level at admission served as the risk factor related to PPDM-A development within 3 years. However, the glucose level at admission lacked significant correlation with PPDM-A in the multivariate model. Lasso regression was employed to eliminate the interference of multiple covariates between admission glucose and glycemic variability, enhancing the credibility of our results. Nevertheless, single-point measurements could not sufficiently indicate the real association of blood glucose with PPDM-A, while LAGE was the dynamic factor overcoming static data limitations. Sensitivity analysis suggested that our results were robust.

This study aimed to focus on clarifying the candidate mechanisms between blood glucose fluctuations and new-onset DM development in AP patients. A plausible explanation for our findings refers to that exposure to fluctuating glucose levels heightens oxidative stress, causing pronounced endothelial dysfunction (38, 39). This, in turn, exacerbates the systemic inflammatory response and insulin resistance, therefore elevating an individual’s risk of developing hyperglycemia in the future (40, 41). Clinically, elevated LAGE (>5.1 mmol/L) predicts hyperglycemic excursions (>4.4 mmol/L) and hypoglycemic risk (>3.48 mmol/L), while inversely associating with time-in-range in type 2 diabetes (42–44).

The association between PPDM-A and elevated serum LDH levels can be attributed to several factors. AP results in extensive tissue damage and an inflammatory response, causing the release of LDH from damaged cells into the blood. Hypoxia and metabolic disturbances during pancreatic inflammation further exacerbate tissue damage, which can increase LDH levels. Elevated LDH levels also reflect the severity of pancreatic necrosis and inflammation, which are related to pancreatic beta-cell dysfunction and increased insulin resistance, leading to diabetes (45).

In addition to LAGE and LDH, increased IL-6 and CRP were also significantly associated with the PPDM-A development. This aligns with research findings indicating that increased IL-6 levels after AP are associated with a higher risk of hyperglycemia (46). IL-6 and CRP increase during inflammation, impacting glucose metabolism through insulin resistance (47, 48). This can lead to impaired insulin receptor phosphorylation, inhibiting glycogen synthesis and promoting glucose release (49–51). Further investigations are necessary to understand the link of abnormal glucose metabolism with adipocytokines for better preventive and therapeutic strategies for PPDM-A.

The higher basic platelet level is the candidate indicator for a higher thrombosis risk, enhanced inflammatory response, while decreased vascular endothelial function (52). The increase in platelet count has been shown as a potential predictor for PPDM-A in this study. This is likely resulted from the inflammatory response triggered by AP which can cause insulin resistance and impaired glucose metabolism. The elevated platelet count may reflect the ongoing inflammation and oxidative stress in the body, which are known risk factors associated with diabetes occurrence (53). It is important to monitor patients with elevated platelet counts following AP closely for developing diabetes, as early intervention and management can contribute to preventing or delaying the onset of the disease.

Furthermore, the relationship between AP severity and the risk of PPDM-A requires careful interpretation. Evidence regarding AP severity as a risk factor is mixed, with some studies supporting the link and others finding no significant association(6,7,10,11). In our study, AP severity was significantly associated with PPDM-A in univariate analysis (**Table 2**), and patients in the high LAGE group had a higher incidence of severe AP (**Table 3**). However, AP severity was not retained as an independent predictor in the multivariate model after LASSO regression. We propose a plausible explanation wherein the impact of AP severity on PPDM-A is largely mediated through its effect on LAGE. More intense inflammatory insults and metabolic stress during AP likely predispose patients to greater glycemic excursions. The resulting glucose variability, quantified by LAGE, then acts as a more direct and measurable driver of future diabetes risk. Therefore, these findings suggest that while the initial injury and severity set the stage, the subsequent metabolic and inflammatory dysregulation, particularly glucose variability, may be more proximal determinants of PPDM-A risk.

However, this study had several limitations that need to be acknowledged. First, as a retrospective analysis conducted at a single institution using electronic medical records, this study may be subject to recall and selection biases commonly associated with historically documented patient information. Therefore, the generalizability of the conclusions should be confirmed in future multi-center, prospective studies with larger cohorts. Second, the inability to obtain blood specimens during the follow-up period precluded the assessment of islet function (e.g., by insulin or C-peptide release tests), although such functional evaluations would have substantially strengthened the investigation of PPDM-A’s pathological mechanisms. Third, while in-hospital glucose monitoring enabled the assessment of GV during the acute phase of AP, the absence of glucose monitoring data in the chronic phase limited a comprehensive evaluation of its association with PPDM-A and restricted insights into the temporal dynamics of GV’s impact. In addition, conventional metabolic indices such as BMI and HOMA-IR were not included in our analysis due to concerns regarding their reliability in the acute phase of severe pancreatitis. This decision was based on two main considerations: (1) the impracticality of obtaining accurate anthropometric measurements in critically ill patients, and (2) the substantial confounding effect of acute pancreatitis–related β-cell dysfunction on insulin resistance indices (21–24).

## Conclusion

To conclude, this study indicated that secondary diabetes is prevalent among AP patients. GV exerts a vital role in risk prediction for PPDM-A. Abnormal GV is the efficient way to stratify and manage risk, which can be used to predict the possibility of PPDM-A clinically. More prospective research is needed to clarify the mechanisms of GV promoting the incidence of PPDM-A and to determine whether GV interventions positively influences the improvement of clinical outcome.

## Data Availability

The raw data supporting the conclusions of this article will be made available by the authors, without undue reservation.
