# Supplementary material for: Risk stratification and predictive value of glucose variability for the development of post-acute pancreatitis diabetes mellitus
Source: Front Endocrinol (Lausanne). 2025 Dec 1;16:1501530. doi: 10.3389/fendo.2025.1501530 (PMC12702752; doi:10.3389/fendo.2025.1501530)

Supplemental materials

S Figure 1


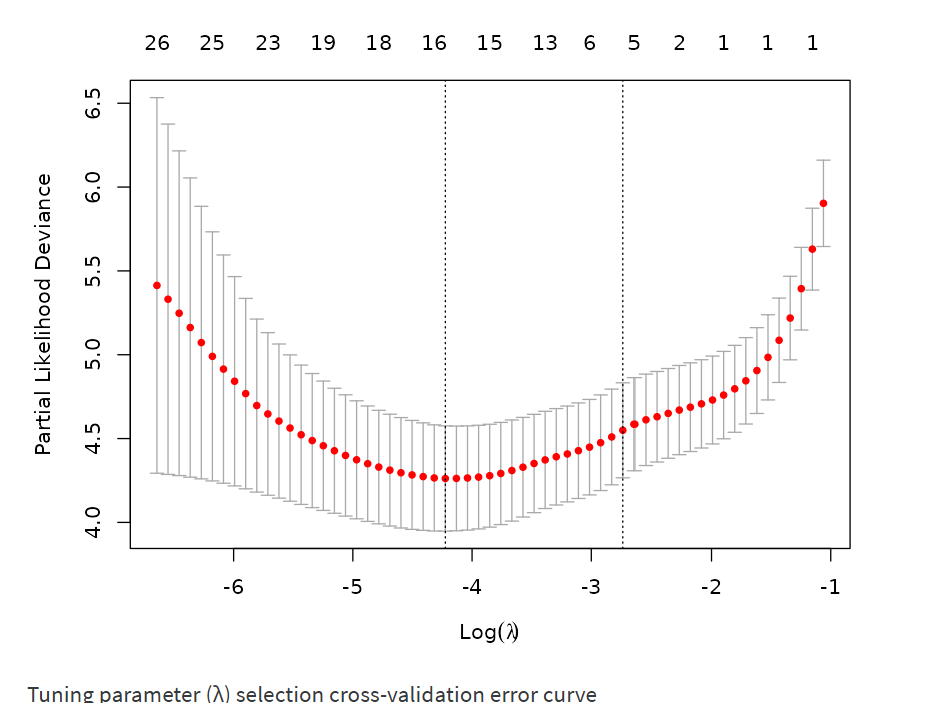


Selection of tuning parameter (λ) in the LASSO regression using 10-fold cross-validation via minimum criteria. The partial likelihood binomial deviance is plotted vs log (λ). At the optimal values log (λ), where features are selected, dotted vertical lines are set using the minimum criteria and the one standard error of the minimum criteria.

S Figure 2


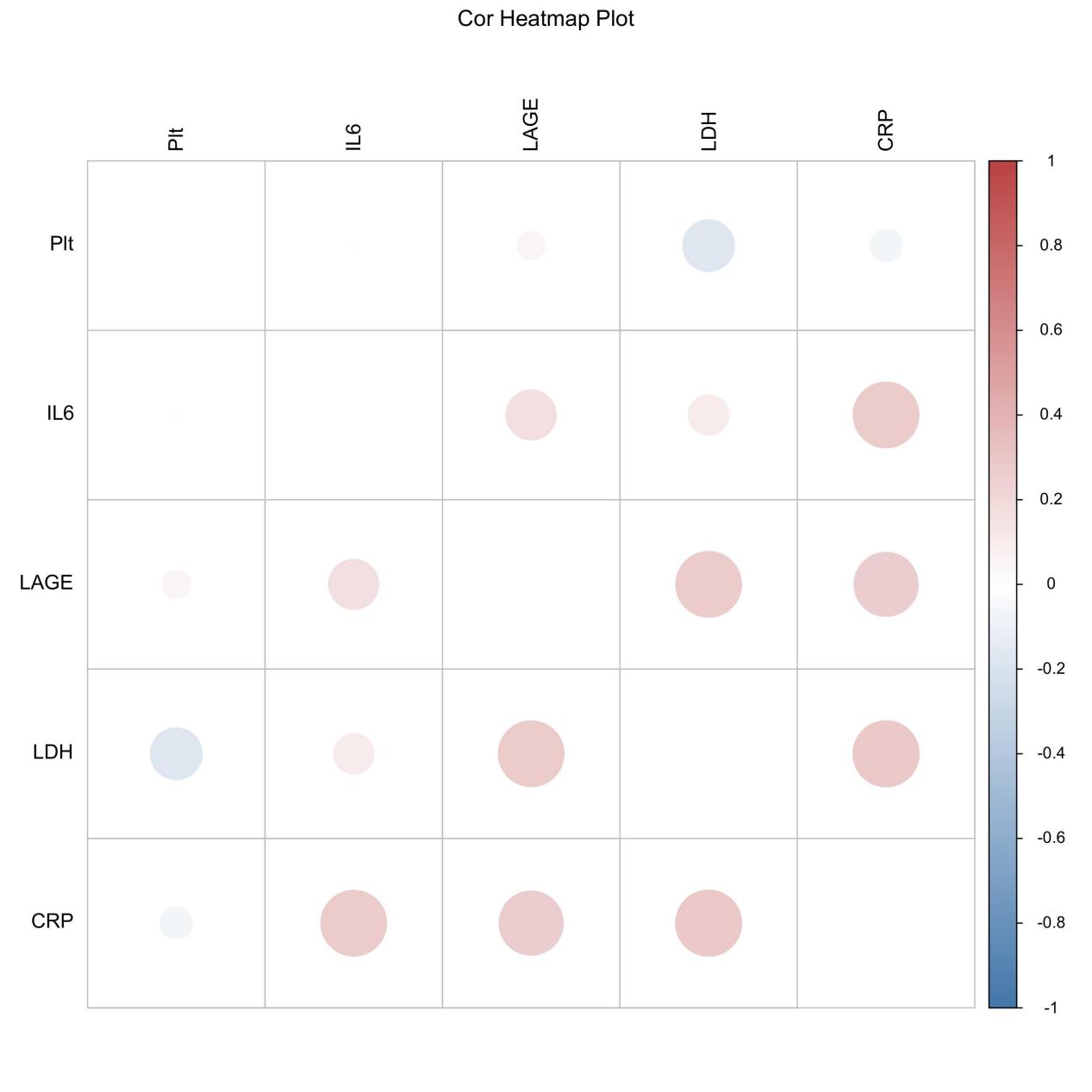


S Figure 3


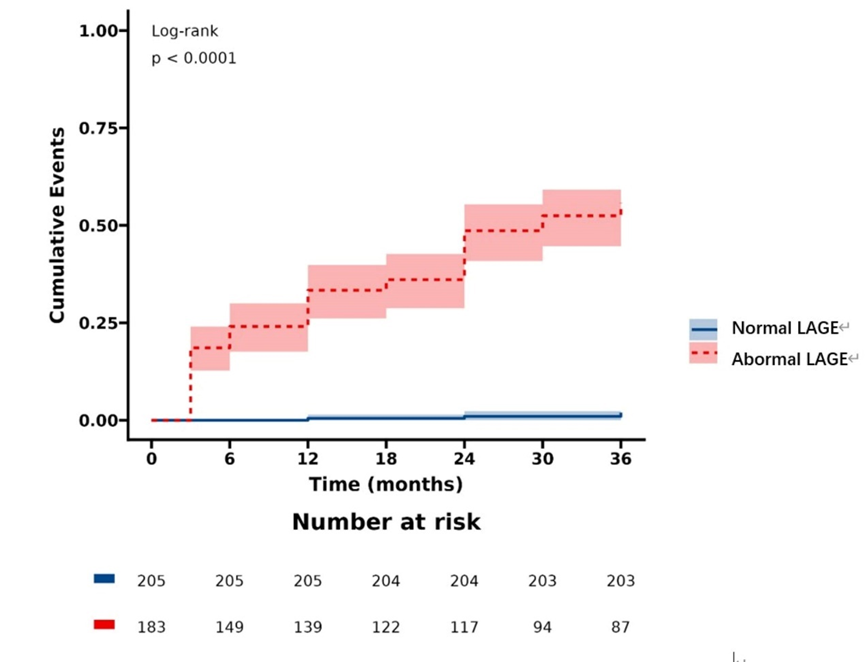

Supplement: Supplementary file 1 [file DataSheet1.docx]
